# Construction of frailty and risk prediction models in maintenance hemodialysis patients: a cross-sectional study

**DOI:** 10.3389/fmed.2024.1296494

**Published:** 2024-10-08

**Authors:** Huan Liu, Mingfen Tao, Man Zhang, Zhiqing Zhou, Yang Ni, Qin Wang, Xiang Zhang, Chenru Chi, Dan Yang, Mengqi Chen, Xiubin Tao, Ming Zhang

**Affiliations:** ^1^Department of Hemodialysis, The First Affiliated Yijishan Hospital of Wannan Medical College, Wuhu, China; ^2^Department of Nursing, Shaanxi Provincial People's Hospital, Xi’an, China; ^3^Department of Nursing, The First Affiliated Yijishan Hospital of Wannan Medical College, Wuhu, China; ^4^Department of Graduate School, Wannan Medical College, Wuhu, China; ^5^School of Educational Science, Anhui Normal University, Wuhu, China; ^6^School of Innovation and Entrepreneurship, Wannan Medical College, Wuhu, China

**Keywords:** frailty, prevalence, hemodialysis patients, multicentre, study

## Abstract

**Objective:**

As the prevalence of diabetic nephropathy and hypertensive nephropathy increases with age in mainland China, the number of patients with end-stage renal disease is increasing, leading to an increase in the number of patients receiving maintenance hemodialysis. Considering the harmful effects of frailty on the health of maintenance hemodialysis patients, this study aims to identify hemodialysis patients at risk for frailty at an early stage, in order to prevent or delay the progression of frailty in the early stage, so as to prevent the adverse consequences of frailty.

**Methods:**

A total of 479 patients admitted to the blood purification centers of two grade tertiary hospitals in Anhui Province, China, using convenient sampling. The Frailty Scale, the SARC-F questionnaire, the Simplified Food Appetite Questionnaire (SNAQ) and the mini nutritional assessment short-form (MNA-SF) were used in the study. Pearson correlation analysis was used to explore the correlation among the frailty influencing factors.

**Results:**

The incidence of frailty was 24.0% among 479 Chinese hemodialysis patients. Gender (*p* < 0.05), Malnutrition (*p* < 0.001), sarcopenia (*p* < 0.001), and feel tired after dialysis (*p* < 0.001) were highly correlated with frailty in Chinese hemodialysis patients. Serum albumin concentration (*p* < 0.05) was a protective factor of frailty.

**Conclusion:**

This survey shows that frailty was highly prevalent among Chinese hemodialysis patients. Medical staff and their families should make early judgments and carry out interventions on the risk of frailty.

## Introduction

Frailty is a clinical syndrome that is marked by a depletion of physical reserves and multiple system disorders. This depletion reduces the body’s ability to cope with stress and maintain homeostasis. However, it also increases the body’s resilience to stressful events and diseases (1). It is crucial for us to recognize that frailty is a dynamic disease which evolves with time and organ dysfunction (2). Several factors like inflammatory markers, hospitalization, and malnutrition were identified as predictors of frailty transitions (3, 4). As China’s population ages rapidly, frailty has become an important and prominent public health problem. With the increasing incidence of end-stage chronic renal failure caused by chronic kidney disease and other chronic diseases, the number of hemodialysis patients in China is also significantly increasing year by year (5).

The number of people underwent renal replacement therapy worldwide was estimated at 262 million in 2010, which is expected to increase to 543.9 million by 2030 (6). Frailty poses a very serious threat to the quality of life and health of the maintenance hemodialysis patients. The incidence rate of frailty in dialysis patients ranges from 14 to 73% (7, 8). Many previous studies reported that frailty was a predictor of adverse outcomes. Clinical outcomes include mortality (9), hospitalization (10), and falls (11). Considering the adverse health outcomes caused by the high incidence and prevalence of frailty and related factors in maintenance hemodialysis patients, should be explored the influencing factors of frailty in maintenance hemodialysis patients, so as to provide a scientific basis for early prevention and intervention. Frailty is divided into three subgroups: social frailty, physical frailty, and cognitive frailty, with sarcopenia being the major component of physical frailty (12). Sarcopenia is defined as a muscle disorder (13, 14) characterized by a gradual and generalized loss of muscle strength and loss of muscle mass (15, 16). Sarcopenia is associated with osteoporosis (17), falls (18), functional disability, death, and other adverse outcomes (19). Studies had shown that sarcopenia could reduce the strength of swallowing muscles (20–22), which was risk factors for dysphagia. Sarcopenia has become an important predictor of frailty in hemodialysis patients. However, the relationship between sarcopenia status and frailty status in maintenance hemodialysis patients is still neglected. Therefore, it is crucial to consider the impact of maintenance hemodialysis patients’ sarcopenia on frailty.

Appetite is the state of motivation to eat (23), which is affected by many factors (24). Studies have found that appetite lose is associated with poor mental health (25), decreased quality of life (26), hospitalization (27), and increased mortality (28). Appetite change is one of the symptoms associated with uremia (29). Appetite loss occurs frequently in patients undergoing maintenance hemodialysis (HD). Uremia may cause loss of appetite by changing the levels of leptin, ghrelin, and neuropeptide Y, etc. (30). Appetite loss is associated with an increased risk of death, increased hospitalization, and poorer quality of life (31). When maintenance hemodialysis patients develop a poor appetite for some time, they may become frail through malnutrition, which may harm their health. Although the important role appetite plays in the frailty of maintenance hemodialysis patients, it remains an under-studied variable, especially in China.

Malnutrition is a clinical syndrome caused by nutritional imbalance (deficiency or excess) with measurable adverse effects on body tissue/morphology (body type, size, composition) or function and/or clinical outcome (32). Malnutrition can be divided into iatrogenic and non-iatrogenic (33). The iatrogenic factor is caused by dialysis treatment. Nutrient losses during dialysis range from 3 to 8 g of amino acids and 3–9 g of protein per day. Non-iatrogenic factors are spontaneously produced by loss of appetite and decreased physical function. Inoue et al. (34) and Goisser et al. (35) found that malnutrition assessed by MNA-SF or MNA-FF was a significant predictor of ADL improvement at discharge and 6 months postoperatively. Miu and Lam (36) reported higher in-hospital mortality in malnourished patients as assessed by MNA-SF compared with high-risk and well-nourished patients. Previous evidence suggests that malnutrition leads to a loss of muscle mass and strength, which can contribute to the onset of sarcopenia and subsequent physical damage, both of which are important factors in frailty (37). A meta-analysis of older adults found that the prevalence of malnutrition in older adults might range from 6% (95% CI, 4.6–7.5) to 29.4% (95% CI, 21.7–36.9) (38). And, Wojzischke et al. (39) reported approximately 47% of elderly rehabilitation patients were at the risk of malnutrition. Due to its clinical impact on acute and chronic disease, malnutrition is widely recognized as being associated with poor health (40, 41).Furthermore, malnutrition is now recognized as one of the most important modifiable prognostic factors, which worsens prognosis and mortality in elderly patients (42, 43). Malnutrition is also the risk factor for osteoporosis, sarcopenia, and frailty (44). Because of the detrimental effects of malnutrition on the musculoskeletal system, malnourished patients should be screened for sarcopenia and frailty syndromes, and synergies between therapeutic interventions should be enhanced (45). Malnutrition impairs normal brain function and promotes cognitive decline (46). Furthermore, the central role of malnutrition in the pathophysiology of frailty and sarcopenia is well established (47).

Predictive models can make the most accurate predictions possible by learning from data and can be used to help select prevention and treatment strategies (48). Nevertheless, the present research on frailty in hemodialysis patients in China was limited to current status surveys, and no effective frailty risk identification tool was constructed for hemodialysis patients. Therefore, this study aims to explore the incidence of frailty in hemodialysis patients and its influencing factors and construct a risk prediction nomogram model based on easily available predictors to provide a reference for the early identification of patients with frailty.

## Materials and methods

### Study design

This multicentre cross-sectional study was conducted at the blood purification centers of two grade A tertiary hospitals in Anhui Province, China, using convenient sampling. The questionnaire and study data were strictly confidential and used only for this study.

### Clinical data

Participants strictly meet the following selection criteria: (a) Age ≥ 18 years; (b) Dialysis for more than 3 months; (c) Maintenance hemodialysis (MHD) treatment 2–3 times a week; (d) Normal comprehension; and (e) obtaining the informed consent and voluntary participation of the participants. Exclusion criteria included (a) inpatient MHD during the investigation period; (b) cognitive impairment or mental illness; (c) having serious complications from hemodialysis or suffering from other serious physical diseases and unable to take care of themselves; And (d) refused to participate in the study. Of the 500 maintenance hemodialysis patients who met the inclusion criteria who were invited to participate, 21 eligible participants declined to participate due to low level of compliance. The remaining 479 participants returned complete and valid questionnaires with a valid sample size of 479 (participation rate = 95%).

### Data collection

**Figure 1:**
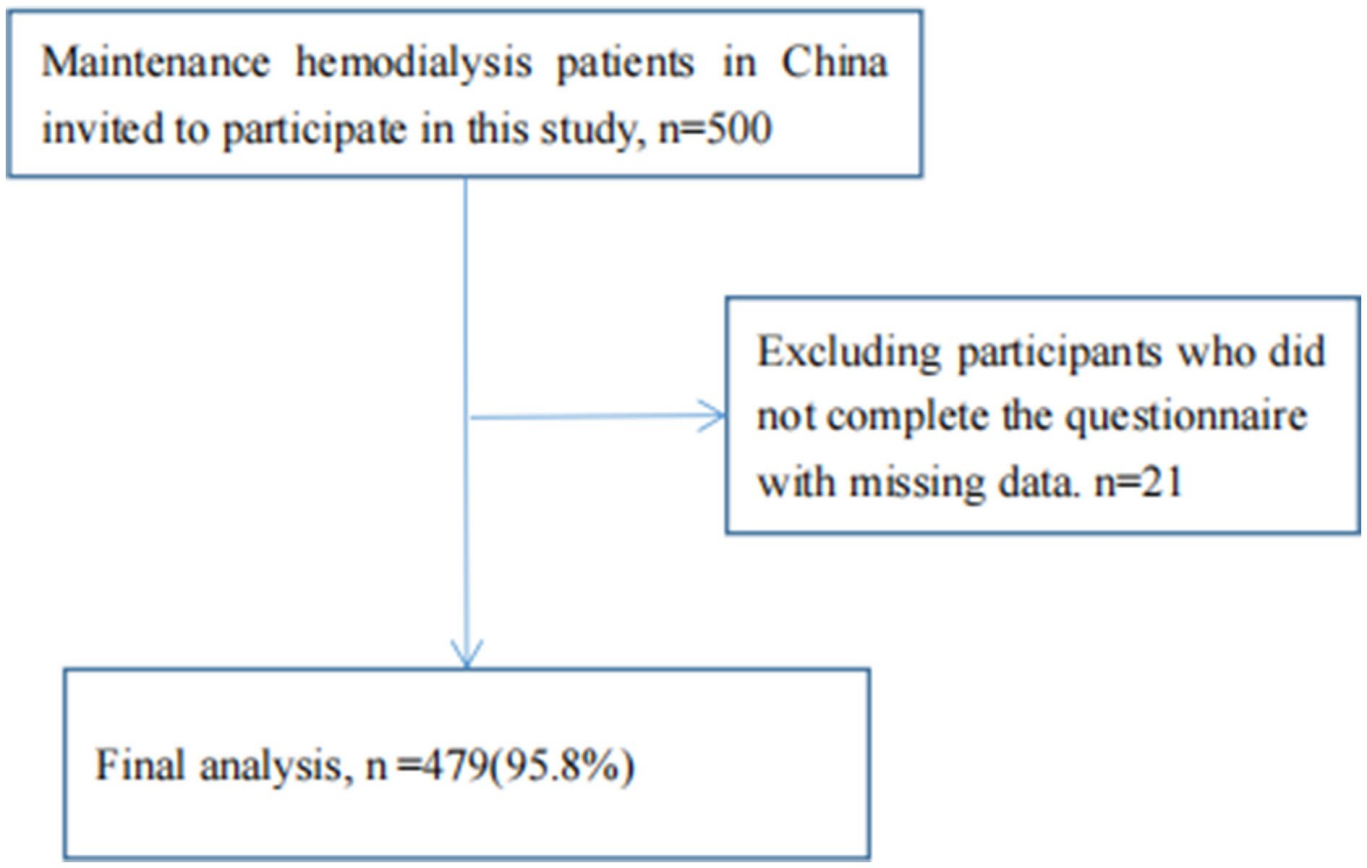
Sample selection process for this cross-sectional study.

The time for participants to complete the survey was approximately 5–10 min. Four trained nursing graduate students supervised and coordinated the completion of the questionnaire. The investigators received unified training from the members of the research group. Prior to the survey, maintenance hemodialysis patients were informed that the study was conducted under the principle of complete anonymity and strict confidentiality, and that patients could only participate in the study by voluntarily signed informed consent. In addition, the investigators introduced the purpose and significance of the study to the maintenance hemodialysis patients. A total of 500 maintenance hemodialysis patients completed the investigation, of which 21 were excluded, and the remaining 479 respondents met the requirements, with an effective response proportion of 95.8% (details are shown in Figure 1).

### Instruments

#### Sociodemographic variables

The demographic questionnaire was designed by the first author after literature search and according to the purpose of the study, and collected information such as age, residence, gender, marital status, education level, the times of hemodialysis per week, the duration of hemodialysis, and type of medical insurance, were collected by investigators through face-to-face interviews.

#### Frailty scale

Frailty was measured using the Frailty Scale proposed by the International Society of Gerontology Expert Group (1). The scale consists of 5 items, 1 point for answering “yes” and 0 point for answering “no,” the total score ranges from 0 to 5 points, and ≥ 3 points indicate the presence of frailty. The FRAIL scale has also been verified in Chinese maintenance hemodialysis patients (49). In this study, Cronbach’s alpha coefficient for the FRAIL scale was 0.843.

#### The SARC-F questionnaire

The SARC-F is a simply, rapidly and conveniently screening questionnaire for rapidly identifying of individuals at risk of sarcopenia, and both the updated EWGSOP consensus and the AWGS consensus recommend the SARC-F questionnaire as a tool for screening patients for sarcopenia (50, 51). The standard SARC-F scale examines five domains: (1) strength, (2) assistance with walking, (3) rising from a chair, (4) climbing stairs, and (5) falls. Each of the first four questions on the sarcopenia scale provides three possible answers: “no problem,” “some problems,” and “a lot of problems or impossible.” For the last question, falls, the possible answers were “never,” “one to three times,” and “four or more times.” Each item was scored with 0, 1, and 2 points, respectively. The Cronbach’s alpha coefficient of this Sarcopenia scale is 0.87, which has relatively high reliability and validity.

#### Appetite loss

Appetite loss was tested using the Brazilian version of the Simplified Food Appetite Questionnaire (SNAQ) (52). The SNAQ is a simple Likert-type questionnaire with the following questions: (i) My appetite is very poor; poor; average; good; very good. (ii) When I eat, I feel full after a few bites; I feel full after about a third of a meal; I feel full after half a meal; I feel full after eating; I almost never felt full. (iii) I am rarely hungry; occasionally; sometimes; mostly; all the time. (iv) Food tastes very bad; bad; average; good; very good. The total score is ranging from 4 to 20 points. The lower the score, the worse the appetite. A score of 14 or lower indicates the risk for anorexia (53). The Cronbach’s alpha coefficient of this SNAQ is 0.835.

#### Malnutrition

The nutritional status of patients was assessed using the mini nutritional assessment short-form (MNA-SF) (54). Including 6 items: food intake and food intake reduction in the past 3 months, weight change, activity ability, acute disease or psychological trauma, mental and psychological problems, and body mass index. The total score is 14 points, ≤7 points means malnutrition. It has been verified that MNA-SF is suitable for the nutritional assessment of elderly hospitalized patients with chronic diseases (55). The Cronbach’s alpha coefficient of this MNA-SF is 0.91.

### Statistical analysis

The results of the survey were entered into the questionnaire Star software by two surveyors. The statistical analyses were conducted with STATA 15.0 (StataCorp. College Station, TX, USA). All tests were two-sided and a *p* < 0.05 was used to determine statistical significance. The results of the Kolmogorov–Smirnov test indicated that the data were normally distributed. In the study, categorical variables were represented using numerical values and percentages, while continuous variables were presented using the mean value and standard deviation. SPSS version 21.0 (IBM Corporation, Armonk, NY, USA) was used for all statistical analyses, and *p* < 0.05 were considered statistically significant. Binary logistic regression analysis was performed to analyze the factors associated with frailty, and the odds ratios (ORs) and 95% confidence intervals (CIs) were calculated.

## Results

### Participant characteristics

As shown in Table 1, a total of 479 patients with maintenance hemodialysis were included in this study. Among 479 participants included in the data analysis, the age of the respondents ranged from 20 to 91 years old, which the mean age being (59.36 ± 18.24) years old. A total of 246 (51.4%) were male, and 233 (48.6%) were female. A total of 215 (44.9%) live in the rural, 108 (22.5%) live in the town, and 156 (32.6%) live in the city. A total of 383 (80.0%) of the participants had been hospitalized in the past 6 months, and 96 (20.0%) had not. A total of 302 (63.0%) of the participants had no symptoms of fatigue after dialysis, and 177 (37.0%) of the participants had. A total of 273 (57.0%) of the participants felt lonely, and 206 (43.0%) did not feel lonely. A total of 274 (57.2%) of the participants had comorbidities, and 205 (42.8%) of the participants had no comorbidities. Further socio-demographic information about this study is displayed in Table 1.

**Table 1:** The socio-demographic characteristics of the participants (*N* = 479).

Variables	***N*(%)**
Gender	
Male	246(51.4)
Female	233(48.6)
Place of residence	
Rural	215(44.9)
Town	108(22.5)
City	156(32.6)
Age	
20–30 years	40(8.4)
31–40 years	78(16.3)
41–50 years	160(33.4)
51–60 years	101(21.1)
61–70 years	83(17.3)
>70 years	17(3.5)
Hospitalized in the past 6 months	
No	383(80.0)
Yes	96(20.0)
Dialysis frequency	
2 times/week	48(10.0)
3 times/week	431(90.0)
Employment status	
No	432(90.2)
Yes	47(9.8)
Frequency of physical exercise per week	
0 times/week	202(42.2)
1–2 times/week	146(30.5)
≥3 times/week	131(27.3)
Marital status	
Single	46(9.6)
Married	399(83.3)
Divorced	16(3.3)
Others	18(3.8)
Education	
Elementary school and below	241(50.3)
Junior high school	135(28.2)
High school	62(12.9)
Junior college	31(6.5)
College degree and above	10(2.1)
Feel tired after dialysis	
No	302(63.0)
Yes	177(37.0)
Feel lonely	
No	206(43.0)
Yes	273(57.0)
Comorbidity	
No	205(42.8)
Yes	274(57.2)
Sleep time per day	
≤5 h/day	79(16.5)
6 h/day	135(28.2)
7 h/day	164(34.2)
8 h/day	83(17.3)
9 h/day	7(1.5)
≥10 h/day	11(2.3)

### Factors associated with frailty in the univariate analysis

In this study, the prevalence of frailty among the Chinese hemodialysis patients was 24.0% (115/479). There were significant differences between malnutrition, appetite, sarcopenia, comorbidity, feel tired after dialysis, sleep time per day, frequency of physical exercise per week, employment status, dialysis frequency, hospitalized in the past 6 months, age, place of residence, gender, (*p* < 0.05, Table 2).

**Table 2:** Characteristics of the participants based on the presence of frailty (*n* = 479).

Physical symptoms and health status	Non-frailty (*N* = 364)	Frailty (*N* = 115)	Chi-square statistics (X^2^)	*p*-value
	*N* (%)	*N* (%)		
Malnutrition				
Yes	42 (39.6)	64 (60.4)	98.689	<0.001***
Appetite				
Yes	301(73.1)	111 (26.9)	13.892	<0.001***
Sarcopenia				
Yes	31 (28.2)	79 (71.8)	178.899	<0.001***
Comorbidity				
Yes	198 (72.3)	76(27.7)	4.879	0.027*
Feel lonely				
Yes	199 (72.9)	74 (27.1)	3.339	0.068
Feel tired after dialysis				
Yes	122 (68.9)	55 (31.1)	7.681	0.006**
Sleep time per day				
≤5 h/day	40 (50.6)	39 (49.4)	37.138	<0.001***
6 h/day	103 (76.3)	32 (23.7)		
7 h/day	135(82.3)	29(17.7)		
8 h/day	72(86.7)	11(13.3)		
9 h/day	6(85.7)	1(14.3)		
≥10 h/day	8(72.7)	3(27.3)		
Education				
Elementary school and below	171 (71.0)	70 (29.0)	7.827	0.098
Junior high school	112 (83.0)	23 (17.0)		
High school	49(79.0)	13(21.0)		
Junior college	25(80.6)	6(19.4)		
College degree and above	7(70.0)	3(30.0)		
Marital status				
Single	39 (84.8)	7 (15.2)	3.190	0.363
Married	300 (75.2)	99 (24.8)		
Divorced	13(81.3)	3(18.8)		
Others	12(66.7)	6(33.3)		
Frequency of physical exercise per week				
0 times/week	128(63.4)	74 (36.6)	33.760	<0.001***
1–2 times/week	118(80.8)	28 (19.2)		
≥3 times/week	118(90.1)	13(9.9)		
Employment status				
Yes	42 (89.4)	5 (10.6)	5.106	0.024*
Dialysis frequency				
2 times/week	42 (87.5)	6 (12.5)	3.873	0.049*
3 times/week	322 (74.7)	109 (25.3)		
Hospitalized in the past 6 months				
Yes	62 (64.6)	34 (35.4)	8.565	0.003**
Age				
18–30	33 (82.5)	7 (17.5)	27.084	<0.001***
31–40	70 (89.7)	8(10.3)		
41–50	128(80.0)	32(20.0)		
51–60	73(72.3)	28(27.7)		
61–70	52(62.7)	31(37.3)		
>70	8(47.1)	9(52.9)		
Place of residence				
Rural	153 (71.2)	62 (28.8)	9.585	0.008**
Town	79 (73.1)	29 (26.9)		
City	132 (84.6)	24 (15.4)		
Gender				
Male	202(82.1)	44(17.9)	10.390	0.001***
Female	162(69.5)	71(30.5)		

*p < 0.05, **p < 0.01, ***p < 0.001.

### Factors associated with frailty

Factors affecting frailty of the Chinese hemodialysis patients are shown in Table 3. When Chinese hemodialysis patients with malnutrition (OR = 4.557, 95% CI 2.474–8.393), sarcopenia (OR = 18.171, 95% CI 9.811–33.655), male (OR = 2.458, 95% CI 1.354–4.462), feel tired after dialysis (OR = 3.034, 95% CI 1.657–5.556), the risk of frailty was greater. Serum albumin concentration was a protective factor for frailty (OR = 0.866, 95% CI 0.797–0.941).

**Table 3:** Binary logistic regression analysis of factors associated with frailty.

Variable	B	SE	Wald	*P*	OR	95% CI
Malnutrition	1.517	0.312	23.689	0.000	4.557	2.474–8.393
Sarcopenia	2.900	0.314	85.041	0.000	18.171	9.811–33.655
Gender	0.899	0.304	8.732	0.003	2.458	1.354–4.462
Feel tired after dialysis	1.110	0.309	12.925	0.000	3.034	1.657–5.556
Serum albumin concentration	−0.144	0.042	11.518	0.001	0.866	0.797–0.941
Constant	2.293	1.672	1.881	0.170	9.904	

### Construction of a frailty prediction model for Chinese hemodialysis patients

**Figure 2:**
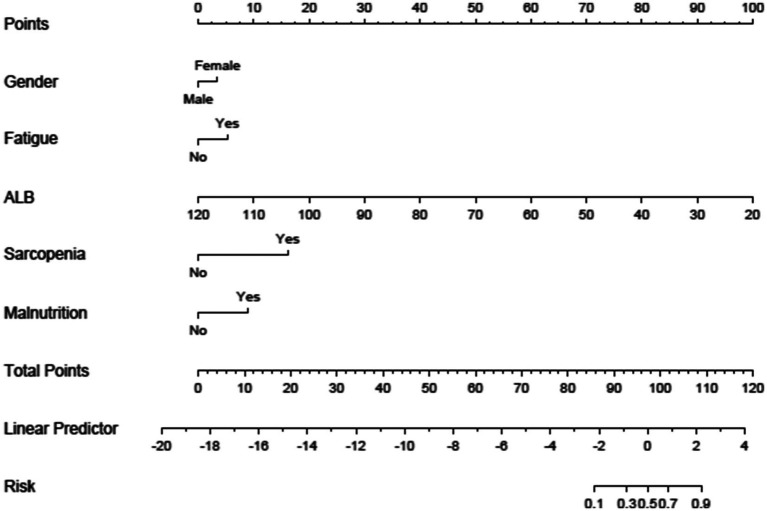
Prediction model of frailty among Chinese haemodialysis patients.

A nomogram model for the risk of frailty in patients with chronic diseases was established based on the independent influencing factors screened out by binary logistic regression. The nomogram model included scores, five independent influencing factors (gender, fatigue, ALB, sarcopenia, and malnutrition), total score, and probability of frailty occurrence, see Figure 1. Figure 2 shows the corresponding Points when the independent variables of gender, fatigue, ALB, sarcopenia, and malnutrition take different values.

### Predictive model validation

#### Discrimination

**Figure 3:**
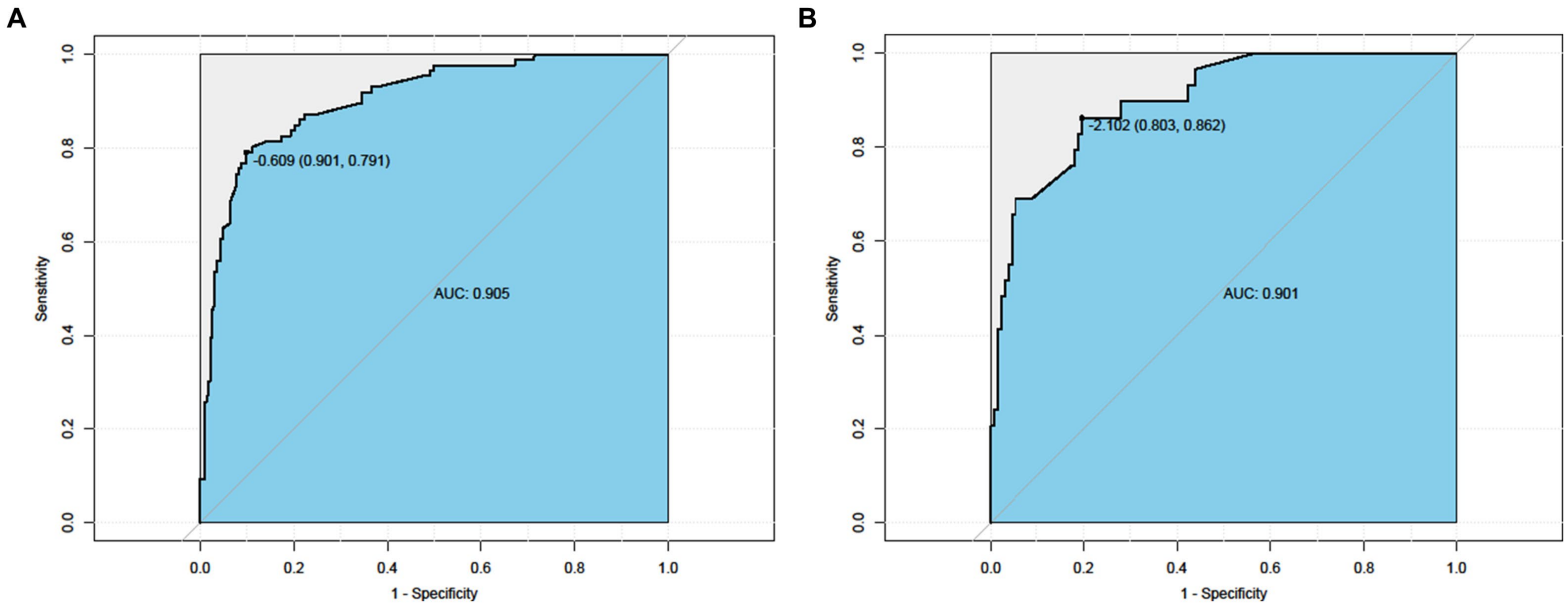
ROC curve. Left Nomogram ROC curves generated from the training dataset. Right Nomogram ROC curves generated using the validation dataset.

AUC values were calculated to assess the discriminative power of the predictive model by examining the prevalence of frailty among Chinese hemodialysis patients in the training and validation sets. As shown in Figure 3, the ROC curves of the training set and the test set, respectively. Taking the training set ROC curve (left picture) as an example, the abscissa is 1-specificity, which is the false negative rate; the ordinate is the sensitivity, which is the true positive rate. The 45° black dotted line represents the reference line, and the solid black curve represents the ROC curve. The results show that the sensitivity of the training set is 0.791, the specificity is 0.901, and the area under the AUC curve is 0.905. The sensitivity of the test set is 0.862, the specificity is 0.803, and the area under the AUC curve is 0.901, indicating that the accuracy of both the training set and the test set is good.

#### Calibration of the predictive model

**Figure 4:**
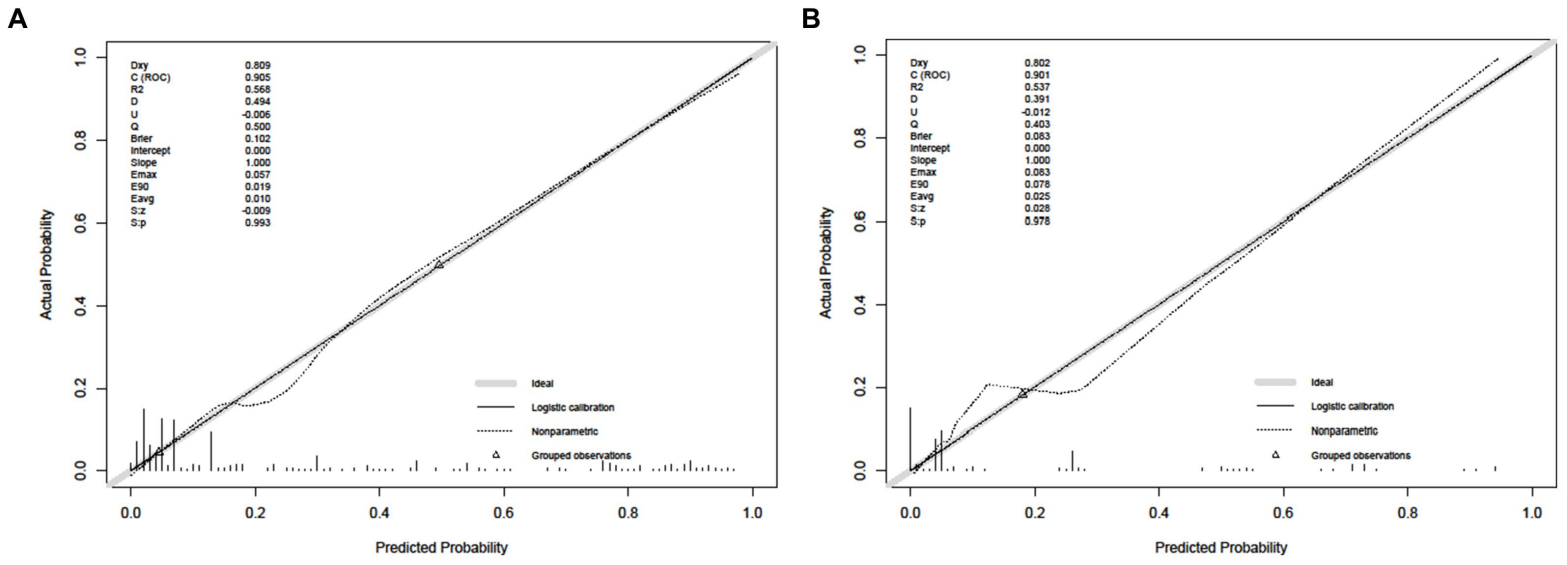
Calibrate curve. **(A)** Calibration plot for the training dataset. **(B)** Calibration plot for the validation dataset.

The Hosmer–Lemeshow test was used to evaluate the goodness of fit of the model. The results showed that the training set *p* = 0.625 > 0.05 and the test set PP = 0.798 > 0.05. It indicates that the model is working well. The calibration curves of the modal plots show that the predicted vulnerability probabilities in the training set (Figure 4A) and validation set (Figure 4B) are in good agreement with the actual vulnerability probabilities.

#### Evaluation of clinical validity

**Figure 5:**
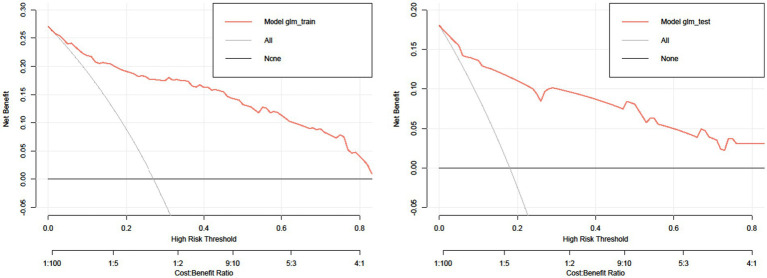
DCA curve.

Figure 5 shows the DCA curves of the training set and test set, respectively. The decision curve shows that the net advantage of the predictive model in the internal validation set is significantly greater than the net advantage in the two extreme cases, indicating that the net advantage of the predictive model and the predictive accuracy nomogram model are better.

## Discussion

There is a growing body of evidence that actual age does not independently predict disease outcomes or death, and that the emergence of the concept of frailty is a more objective and direct response to chronic diseases and the health status and medical needs of older persons (56). The frailty is a systemic change that reduces the ability of the elderly to build up physiological reserves in neuromuscular, metabolic and immune systems for multisystem functional decline. This reduces their ability to fight stress and significantly increases the risk of adverse events (57).

Finally, as expected, sarcopenia was independently associated with frailty in our population. Previous evidence (37) suggests that malnutrition leads to a loss of muscle mass and strength, which can contribute to the onset of sarcopenia and subsequent physical damage, both of which are important factors in frailty. The present study has demonstrated that hemodialysis patients with sarcopenia were two times more likely to develop frailty than those without sarcopenia (58). Furthermore, metabolic acidosis, uremia toxin accumulation, and chronic catabolism have been identified as causes of imbalance in protein production and breakdown in hemodialysis patients (59).

Using correlation, our study showed that appetite factors were closely related to frailty in maintenance hemodialysis patients. When maintenance hemodialysis patients develop poor appetite for a period of time, they may become frailty through malnutrition. And, nutritional status is a risk factor for frailty in patients with MHD (60). This study showed that the more malnutrition the maintenance hemodialysis patients were, the more their frailty was affected. This confirms our observation from another perspective of nutrition (61). Studies (62) have found that debilitation can be remedied to varying degrees through comprehensive post-assessment interventions, including nutritional and exercise regimens and appropriate vitamin D3 supplementation. Simple exercise, cognitive stimulation, debilitating education and nutritional counseling all improved daily activities and debilitating conditions in a one-arm, prospective, non-randomized intervention study. After 4 months of intervention, the length of stay, medical costs and admission rates for frailty patients were successfully reduced (63).

Serum albumin, a negative acute phase agent and marker of systemic inflammation, has been previously associated with an increased risk of cardiovascular death in several patient subgroups (64–66). Studies have found that albuminemia is an indicator of malnutrition, which is considered an important parameter of frailty, and is associated with poor prognosis in patients with heart disease (67). Serum albumin reflects nutritional status and is closely related to frailty (68, 69). Frail older adults often experience unintentional weight loss, indicating that they are malnourished or at risk for malnutrition (70). This study found that maintenance hemodialysis patients have a higher proportion of frailty, so in clinical practice, biochemical indicators must be measured regularly for hemodialysis patients. At the same time, The SARC-F and the SNAQ used in this study are concise and easy to use. They can also be used regularly as regular scales to grasp the patient’s health status on time. This study draws a frailty risk prediction nomogram for hemodialysis patients based on independent influencing factors selected by binary logistic regression analysis. It verifies the model’s validity and practicality by drawing ROC, calibration, and DCA curves. The results show that the model’s diagnostic performance, calibration, discrimination, and clinical practicability are good.

Limitations, First, the main limitation of the current study was its design. Because it was a cross-sectional study, it was not possible to explore a causal relationship between variables. Second, the limitation of this cross-sectional study is that only three hospitals in Anhui Province were surveyed, which may adversely affect the representativeness of the sample, and the sample size of hemodialysis patients will be further expanded to investigate the frailty of patients in different regions of Anhui Province. Third, other objective factors that may be associated with frailty in hemodialysis patients, including inflammatory markers or dialysis adequacy, were not included in this cross-sectional study.

## Conclusion

In conclusion, the incidence of frailty in hemodialysis patients in this study is high. Malnutrition, sarcopenia, gender, and feeling tired after dialysis are independent influencing factors for frailty in hemodialysis patients. The risk prediction model was constructed based on the above-influencing factors. It has good predictive value and is helpful for clinical early screening and early intervention of frailty in hemodialysis patients to reduce adverse outcomes.

## Data Availability

The raw data supporting the conclusions of this article will be made available by the authors, without undue reservation.
